# Virtual Reality-Based Exercise with Exergames as Medicine in Different Contexts: A Short Review

**DOI:** 10.2174/1745017901915010074

**Published:** 2019-07-26

**Authors:** Marcos Túlio Silva Costa, Lanna Pinheiro Vieira, Elizabete de Oliveira Barbosa, Luciana Mendes Oliveira, Pauline Maillot, César Augusto Otero Vaghetti, Mauro Giovani Carta, Sérgio Machado, Valeska Gatica-Rojas, Renato Sobral Monteiro-Junior

**Affiliations:** 1 Department of Internal Medicine, Faculty of Medicine, State University of Montes Claros, Montes Claros, Brazil; 2 Department of Physical Education, State University of Montes Claros, Montes Claros, Brazil; 3 Departament of Neurology, Faculty of Medicine, Universidade Federal Fluminense, Niterói, Rio de Janeiro, Brazil; 4 UFR de Sciences et Techniques des Activités Physiques et Sportives de Paris, Université Paris Descartes, Paris, France; 5 Faculty of Physical Education, Universidade Federal de Pelotas, Pelotas, Rio Grande do Sul, Brazil; 6 Departament of Public Health, University of Cagliari, Cagliari, Italy; 7 Departament of Physical Activity Science, Universidade Salgado de Oliveira, Niterói, Rio de Janeiro, Brazil; 8 Human Motor Control Laboratory, Universidad de Talca, Talca, Chile

**Virtual Reality-Based Exercise with Exergames as Medicine in Different Contexts: A Short Review**

Clinical Practice & Epidemiology in Mental Health, **2019**, 15: 15-20

## Correction

The corrections are provided and replaced online which is mentioned as under:

**Original:**

The name of coauthor was César Augusto Ottero Vaghetti

**Corrected:**

The name of coauthor has been revised as César Augusto Otero Vaghetti

